# Protocol: Pentoxifylline optimal dose finding trial in preterm neonates with suspected late onset sepsis (PTX-trial)

**DOI:** 10.1186/s12887-021-02975-8

**Published:** 2021-11-18

**Authors:** Serife Kurul, H. Rob Taal, Robert B. Flint, Jan Mazela, Irwin K. M. Reiss, Karel Allegaert, Sinno H. P. Simons

**Affiliations:** 1 Department of Pediatrics, Division Neonatology, Research Neonatology (Sk-4246), Erasmus Medical Center, PO Box 2060, Rotterdam, 300 CB The Netherlands; 2 Department of Pharmacy, Erasmus University Medical Center, Rotterdam, The Netherlands; 3 Department of Neonatology, Poznan University of Medical Sciences, Poznań, Poland; 4 Department of Development and Regeneration and Department of Pharmaceutical and Pharmacological Sciences, KU Leuven, Leuven, Belgium

**Keywords:** Neonatal intensive & critical care, Neonatology, Paediatric infectious disease & immunization

## Abstract

**Background:**

Late onset sepsis is a leading cause of death and morbidity in preterm infants. Despite optimal antibiotic treatment, sepsis related mortality and morbidity is still high. Pentoxifylline (PTX) is a methylxanthine with promising immunomodulatory properties, which can be used as an additional therapy next to antibiotics in preterm infants. PTX is increasingly used off-label in neonatal intensive care units, however up till now no dose finding study has been done for PTX in this specific population. The aim of this study (PTX-trial) is to determine the optimal dose of PTX in preterm infants (gestational age < 30 weeks) with (suspected) late onset sepsis. Dose finding in this particular population is unique, since for most drugs used in neonates the optimal dosage has not been investigated in phase II dose-seeking studies.

**Methods:**

The PTX-trial is a prospective open label sequential dose-optimization study with an adapted continual reassessment method. An up-and-down dose-response design will be used, with dose step-up and step-down titration after every 3 patients. The PTX starting dosage will be 30 mg/kg/day in 6 hours as described in most previous neonatal studies. Efficacy is defined by means of biochemical and clinical parameters. Toxicity in these vulnerable patients is unwarranted. The optimal dose is defined as the ED75 (i.e., clinically and chemically effective dose for 75% of patients) in preterm neonates with late onset sepsis. We plan to include 30 neonates to determine the optimal dose using this study design. Subsequently, the optimal dose will be validated in 10 additional preterm neonates. In parallel, pharmacokinetics of PTX and its metabolites will be described as well as longitudinal evaluation of metabolomics and proteomics.

**Discussion:**

The study has been approved by the Regional Medical Ethics Board of Erasmus Medical Center University Rotterdam (MEC 2019-0477) and registered at Clinicaltrials.gov (NCT04152980). Results of the main trial and each of the secondary endpoints will be submitted for publications in peer-reviewed journals.

**Trial registration:**

Clinicaltrials.gov, NCT04152980, Registered November 6th, 2019

## Background

Neonatal sepsis is the leading cause of death and morbidity in preterm neonates worldwide [1–3]. The incidence of late onset neonatal sepsis, defined as sepsis onset after 72 hours after birth, is up to 20% among very low birth weight infants (VLBW, birth weight < 1500 gram) in developed countries [3, 4]. Sepsis therapy in preterm neonates consists of antibiotic treatment, support of vital functions and comfort [5]. Despite adequate antibiotic treatment many preterm neonates do not recover from sepsis, with overall mortality rates of late onset sepsis around 18%, and up to 36% in preterm neonates infected with gram negative organisms [3]. An excessive neonatal inflammatory response with sepsis is associated with increased mortality, but also major morbidity characteristics such as bronchopulmonary dysplasia (BPD), necrotizing enterocolitis (NEC), retinopathy of prematurity (ROP) and cerebral palsy (CP) [3]. In humans, maturation of the adaptive immune system occurs after birth, thus making the innate immune system largely responsible for protection against micro-organisms in the first weeks. Preterm birth results in an additional reduced ability to respond to pathogens characterized by reduced expression of surface innate immune receptors and immature intracellular downstream responses. A consequently reduced anti-inflammatory response and dysregulation of the immune system plays a major role in organ injury in these preterm infants [6, 7].

Pentoxifylline (PTX), a methylxanthine, is currently registered only for peripheral artery disease treatment in adults and is used off label in newborn infants with sepsis. PTX is a promising therapeutic compound, which can be repurposed as adjuvant therapy next to antibiotics for preterm infants with sepsis [8]. PTX acts as a cAMP-phosphodiesterase inhibitor that suppresses TNF-α and modulates important parts of the inflammatory response. Also, the production of other inflammatory cytokines is reduced, such as IL-1α, IL-6, and IL-8, by phagocytes [9] and their subsequent effects are prevented, such as leukocyte adherence, migration, and degranulation [10]. Furthermore, PTX has beneficial effects on endothelial cell function and coagulation in sepsis and exerts its cellular effects on erythrocytes, platelets, endothelial cells, polymorphonuclear leukocytes, macrophages, and fibroblasts [11]. Next to its action on the inflammatory response, PTX also has been suggested to improve the microcirculation [12], one of the key features of sepsis. Given these mechanisms of PTX, it is a high potential candidate for adjuvant immunomodulatory therapy in neonatal sepsis.

PTX has already been shown to have beneficial effects in humans, especially in preterm infants [13–16] in a dose of 30 mg/kg in 6 hours. In a recent meta-analysis of six trials that included in total 416 neonates, it was shown that PTX therapy in this dosage regimen was associated with a decrease in all-cause mortality during hospital stay in infants with late onset sepsis (risk ratio of 0.42) [15]. Furthermore, no side effects have been reported due to PTX treatment in neonates. Overall, there have been multiple suggestions, including clinical studies, that PTX is well tolerated and might improve the recovery and increase the survival of preterm infants suffering from sepsis [14, 15]. Nevertheless, pharmacokinetic (PK) and pharmacodynamic (PD) data of PTX and its active metabolites (M1, M4, M5) are largely lacking. Consequently, target concentrations in this particular population are yet absent, as well as the effect of certain physiological parameters and maturation on clearance. The maturational effect has shown to have an extensive impact for multiple compounds in neonates [17, 18], but has only been poorly described for PTX in neonates [19].

In our Neonatal Intensive Care Unit (NICU) at the Erasmus MC, Rotterdam, the Netherlands PTX is increasingly used in preterm infants with severe sepsis according to a local clinical sepsis protocol based on recent literature. In some countries, such as Poland and Austria, PTX is already used as standard of care therapy for preterm infants with suspected sepsis. Up until now, most clinical studies with PTX to treat sepsis in neonates used the same dosage of 30 mg/kg/day (5 mg/kg PTX per hour for 6 hours every 24 hours) during 3 consecutive days. Research to define optimal dosages based on precise effects, side-effects and the adequate concentration-effect profile of PTX and its active metabolites in preterm neonates is still missing. In the absence of dose finding data, treatment of these patients with the current dose is potentially suboptimal (either under- or overexposure). Finding the optimal dose of PTX is of utmost importance to potentially increase the effectiveness and safety in preterm infants, which can only be obtained from dose seeking research in this vulnerable population. Thus, a dose optimisation study is the next most mandatory step in the drug research and development process for PTX in neonatal sepsis. Because optimal PTX dosing as well as target concentrations of PTX and its metabolites are lacking, we combined these in a prospective dose finding study. The primary objective is to define the ED75 (i.e., clinically and chemically effective dose for 75% of patients) in preterm neonates with late onset sepsis. Simultaneously, as a secondary objective the PK and PD of PTX and its active metabolites will be investigated.

## Methods/Design

### Study settings

Patient recruitment will be performed in two level IV NICUs (Erasmus University Medical Center – Sophia Children’s Hospital, Rotterdam, the Netherlands and Poznan University of Medical Sciences, Poznań, Poland. Recruitment started in December 2019 and is expected to continue for 24 months.

### Participants

**Fig. 1:**
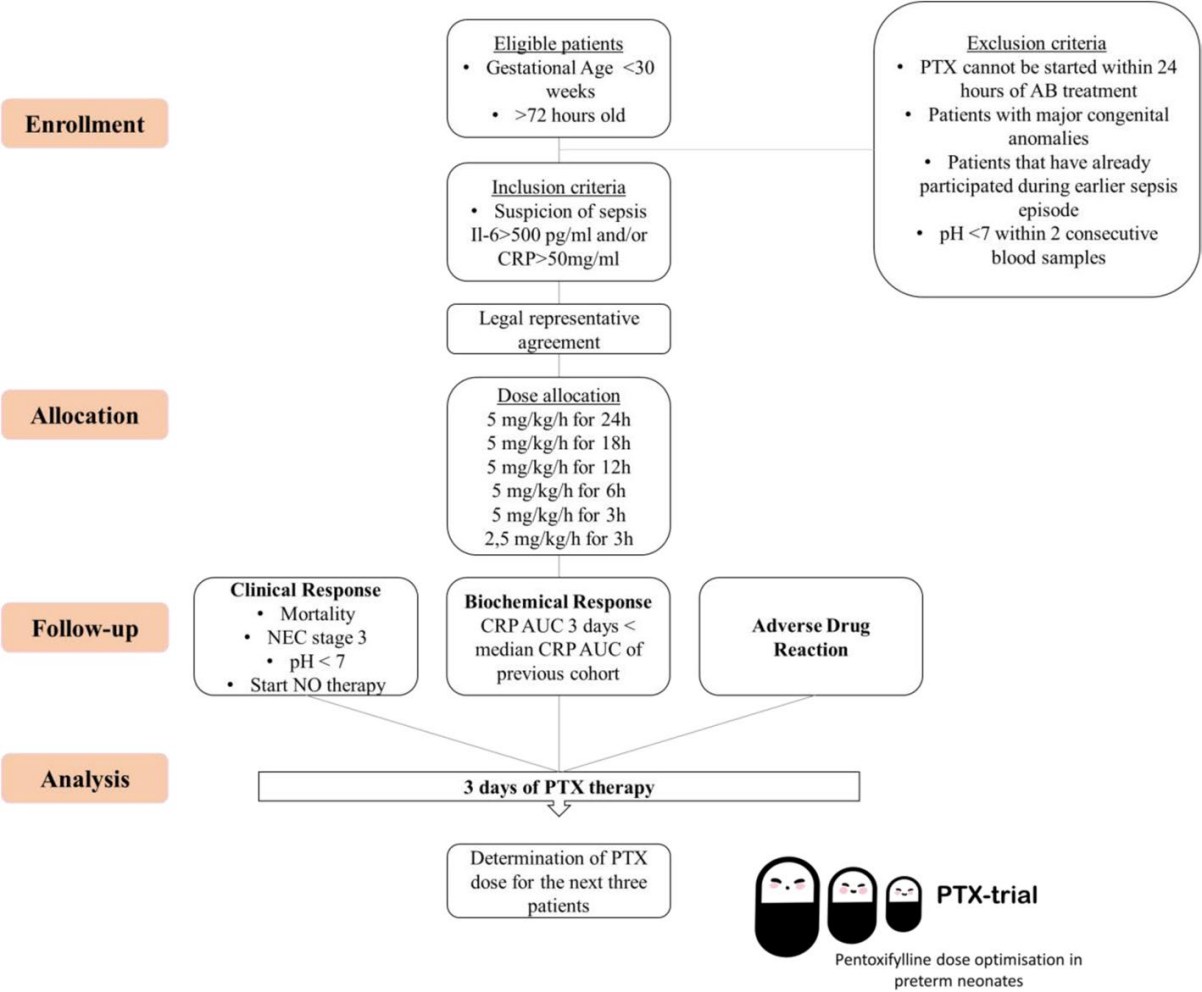
PTX-trial study flowchart. NEC, necrotizing enterocolitis; AUC, area under the curve; AB, antibiotics

Patients are eligible for inclusion if the following criteria are met (1) gestational age below 30 weeks; (2) suspected of sepsis, defined as blood drawn for inflammatory biomarkers and blood culture; (3) increased inflammatory biomarkers at moment of suspicion (C-Reactive Protein (CRP) > 50 mg/L and/or Interleukin-6 (IL-6) > 500 pg/mL); (4) patients with a post-natal age older than 72 hours (late onset sepsis) (see Fig. 1). It is reasonable to assume that patients with (severe) sepsis are most likely to benefit from PTX treatment, and that pharmacokinetics and dynamics are different compared to patients without or with mild sepsis. We aim to investigate the optimal dose in those patients who are likely to benefit the most. Therefore, we have incorporated chemical biomarkers IL-6 and CRP in our inclusion criteria, because these biomarkers are associated with subsequent sepsis severity and can be determined at the moment of sepsis suspicion [20]. By incorporating these biomarkers in our inclusion criteria, they could help select patients with possible culture proven sepsis early in the disease process.

Exclusion criteria concern (1) patients in whom PTX therapy cannot be started within 24 hours of start of antibiotic treatment for any reason; (2) patients with major congenital defects (e.g. congenital heart disease, pulmonary, or gastrointestinal anomalies); (3) patients that already participated in the trial during an earlier episode of late onset sepsis; (4) patients with a pH below 7 in two consecutive blood samples, with at least 1 hour between the blood samples, at start of sepsis episode; and (5) IL-6 values exceeding 25000 pg/mL at time of onset. High IL-6 values represent severe episodes of sepsis and high IL-6 values are associated with high mortality rates [21].

We expect to include 30 neonates to determine the optimal dose using this study design. Planned sample sizes in dose finding studies are generally dictated by practical constraints rather than statistical constraints related to type I error rate or minimum power for testing a specific hypothesis [22, 23]. Generally, cohort sizes in dose finding studies differ between 1-5 patients. A cohort size of 1 is limited by the fact that results could be different by chance, however a cohort consisting of 5 patients will take a long period of time to complete due to the low incidence of severe LONS. In this study we chose for 3 patients in each cohort. Earlier dose finding trials in neonates have shown that a sequential dosing of 10 doses will be sufficient to define an optimal dose, resulting in a total number of 30 neonates to be included. Subsequently, the optimal dose will be validated in 10 preterm neonates.

### Pharmacological intervention

**Fig. 2:**
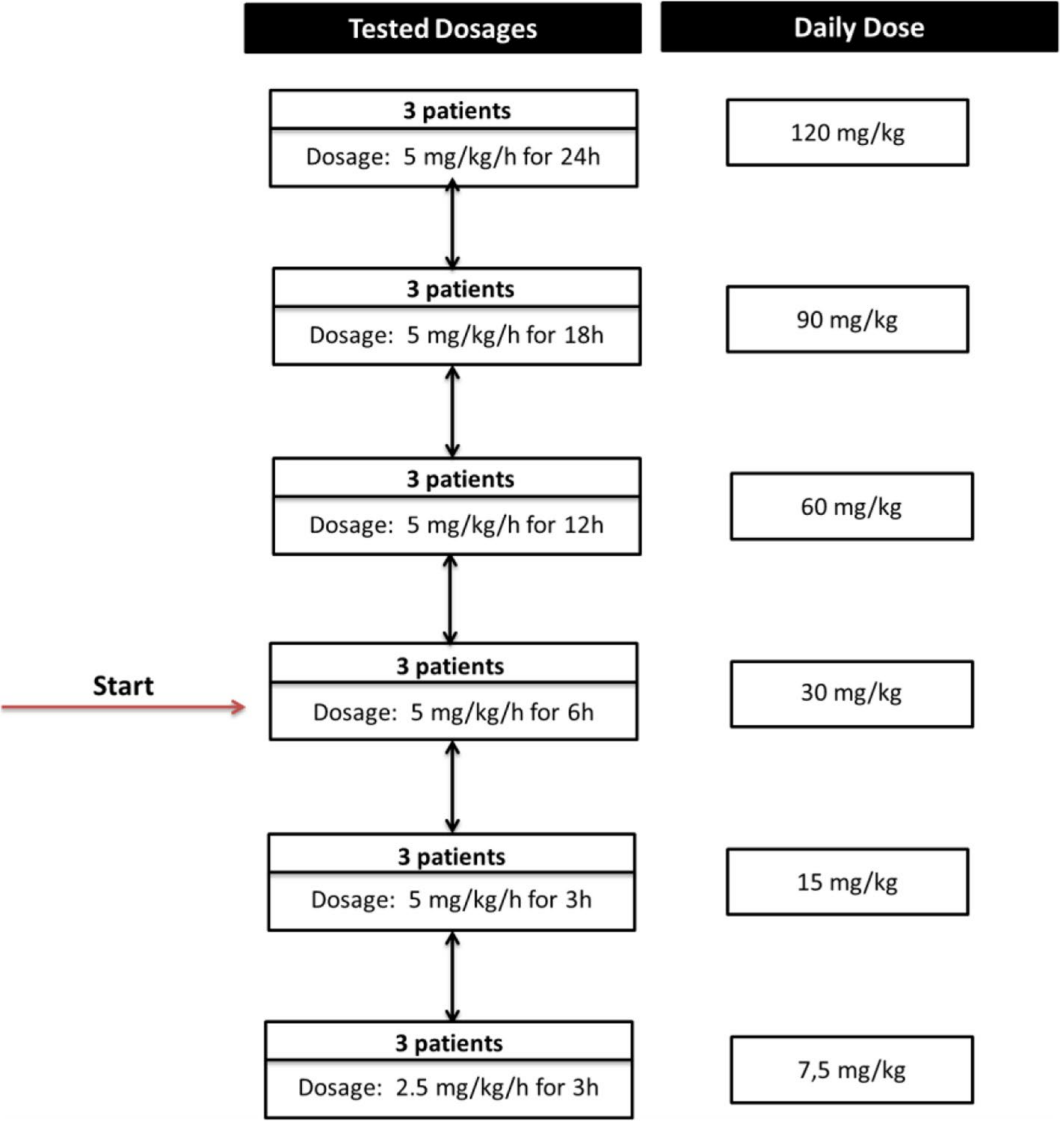
PTX-trial experimental dose-steps of tested dosages

The drug PTX (Trental®, 100 mg/5 ml, Sanofi-Aventis, Austria) will be administered intravenously in a fixed concentration of 5 mg/ml diluted with NaCl 0,9%. The starting dose will be 5 mg/kg/h for 6h every 24 hours for 3-6 days (30 mg/kg/day), which is the most commonly reported dosing regimen in studies until now and has been safely tolerated in these studies, without any reported severe adverse events [16, 24–30]. The duration of therapy will be at least 3 days and can be extended to a maximum of 6 days if judged necessary by the treating physician. In order to find the optimal dose our explicit aim is to study different PTX dosages, next to antibiotics. To do so, dosages will be adjusted with upward and downward dose adjustments (see Fig. 2), based upon clinical and chemical response and adverse drug reactions with decision on the up/downward dose adjustments after each cohort of 3 patients who will receive the same dosage. Intra-individual dose adjustments will not be performed, but the up/down dose adjustments are predefined (Fig. 2).

PTX therapy will be started as soon as possible and ideally concomitant with antibiotic therapy, but no later than 24 hours after start of antibiotic treatment. Based on previous experience PTX seems most effective if initiated in the early stages of sepsis [31]. If administration is delayed until overt septic shock occurs, it may have deleterious effects, such as systemic hypotension [31]. We hypothesise that PTX has a therapeutic window, where no adverse side effects in preterm infants with the previously used dosages have yet been reported.

The maximal dose per hour that will be tested is 5 mg/kg/h continuously, in order to avoid high peak concentrations. It has been predetermined that higher dosages are out of the scope of this trial. This study design does not aim nor allows to explore the highest tolerated dose.

### Primary objective

The principal aim is to determine the ED75, which is defined as a both clinical and chemical effective dose for 75% of patients. The clinical outcome is considered effective when the clinical response to PTX is adequate. Therefore, we have defined a set of clinical endpoints we consider to be inadequate. The biochemical outcome is considered effective if the CRP response is adequate.

In order to asses effectiveness we will determine if the PTX dose is adequate in every individual patient in every cohort of 3 patients who receive the same dosage.

#### An adequate response – criteria for effectivity

An adequate PTX dose is determined by 3 co-primary outcome variables: biochemical response, clinical response and adverse drug reactions. (1) An adequate biochemical response is determined by means of repeated CRP measurement in blood during PTX treatment. We hypothesize that CRP levels will decrease in patients with PTX treatment as was shown in previous studies [16]. CRP levels will be measured in blood at time of onset and 24, 48 and 72 hours after onset. An area under the curve (AUC) will be calculated for each patient. A CRP AUC below the median CRP AUC of the previous cohort considered to be adequate. (2) The clinical response is considered to be inadequate if the following clinical endpoints are reached: mortality, necrotizing enterocolitis Bell stage 3, pH below 7 in 2 consecutive blood samples with at least 1 hour between the 2 samples after start of sepsis treatment and/or the need to start NO therapy with indication pulmonary hypertension after start of sepsis treatment. (3) The dose is considered inadequate if severe adverse drug reactions take place. All adverse events observed spontaneously by the parents or caretakers or observed by the investigator or his staff will be recorded. Potential severe adverse drug reactions will be evaluated by the research group. To assess the causality an algorithm by Du et al [32] will be used. To assess severity the International Neonatal Consortium (INC) Neonatal Adverse Event Scale will be used [33].

**Fig. 3:**
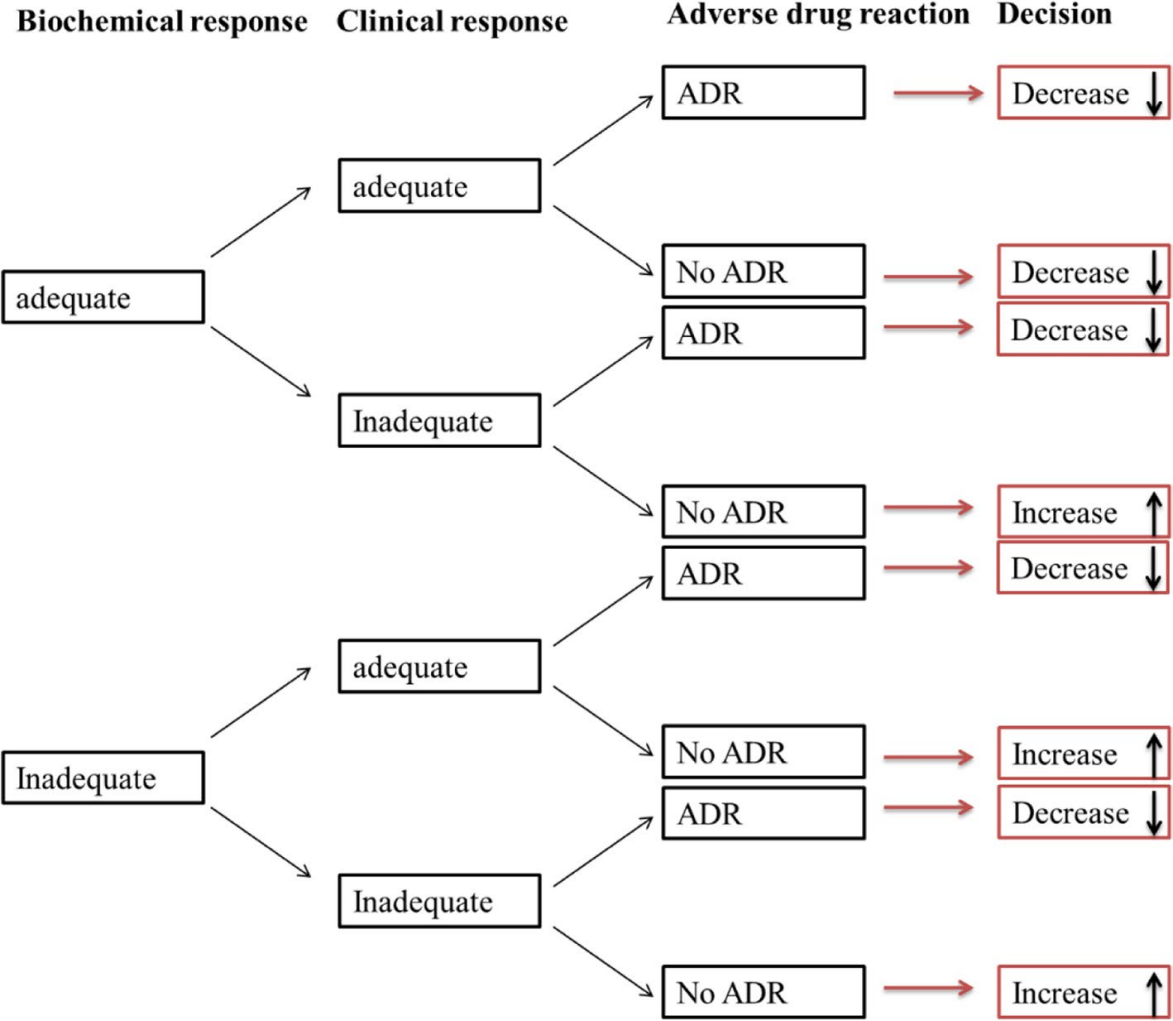
PTX-trial, Clinical decision rule for dose adjustment

After inclusion of each 3 consecutive patients (that receive the same dosage), the efficacy of that specific PTX dose will be analyzed based on clinical effect, biochemical effect and adverse drug reactions. The subsequent dose for the next cohort of 3 patients will be based on the outcome (success/failure) of the previous cohort of patients and decreased/increased in accordance with the predefined dosages (up-and-down method in small samples) [34]. The following dosage for subsequent subjects in this study will be determined by the predefined decision rules (see Fig. 3). The decision rule allows to decrease the dosage when therapy is successful and increase the dosage when therapy fails.

There is a group of 4 neonatologist-researchers (SS, HT, KA, IR) and one pharmacist-researcher (RF) that will be involved in the trial decisions. Minimally 3 of the 5 researchers/clinicians will evaluate the therapy effects independently and make a dose adjustment based on consensus. The decision will be controlled independently by the data-safety monitoring board (DSMB).

If there is a patient in a cohort of 3 patients in whom the decision is to increase the dosage and in the other 2 patients to decrease the dosage and if all patients did not have an adverse drug reaction, the decision for the next cohort of 3 patients will be to decrease the dosage. If there are 2 patients in a cohort of 3 patients in whom the decision is to increase the dosage and in the other patient to decrease the dosage and if all patients did not have an adverse drug reaction, the decision for the next cohort of 3 patients will be to increase the dosage. If in a cohort of 3 patients, one patient has an adverse drug reaction regardless of the decision in the other patients, the decision for the next cohort of 3 patients will be to decrease the dosage. When the patient with the adverse drug reaction is the first or the second in the cohort of three patients, the dose will be adjusted downward immediately for the next cohort of 3 patients. This will also be the case if the lowest tested dosage

If the lowest tested dosage (2.5 mg/kg/h for 3 hours) is reached and the decision would be to decrease the dosage for the next group of patients, a lower dosage won’t be tested. To stimulate the dose optimisation the decision then would be to increase the dosage and after that to follow the decision rule again. Similarly, if the highest dose (5 mg/kg/h for 24 hours) is reached a higher dosage (increase of dose/hour) won’t be tested but the dosage would be decreased after which the decision rule would be followed again. This is based on the hypothesis of our study that the optimal dosage will be between 7.5 mg/kg/day and 120 mg/kg/day.

After the dose-opitimisation trial we will calculate the ED75, the clinical and chemical effective dose for 75% of the patients. The ED75 will be calculated for the pooled clinical and biochemical outcome. As a secondary analysis, we will calculate the ED75 for the clinical and biochemical outcome separately.

### Secondary objectives

#### Pharmacokinetics of PTX and its metabolites

Plasma concentrations of PTX and its metabolites will be measured at sequential time points during treatment. Blood samples can successfully be salvaged after routine clinical tests are complete [35]. Blood sampling concerns either strategic as well as opportunistic sample collection, as long as the allowed total blood volume is not exceeded that corresponds to a negligible additional risk (maximum additional blood sampling of 3% of total blood volume in 4 weeks for research purposes). Each PK sample requires 200 μL of total blood. After centrifugation, the plasma will be harvested and samples will be stored at −80° until measured. Cell pallets will be also be stored for genotyping. We aim to develop a population PK model for PTX and its metabolites using NONMEM® version 7.3 (ICON Development Solutions, Ellicott City, MD, USA), supported by Perl-speaks-NONMEM (PsN®) version 3.4.2 and Xpose version 4.3.5 [36]. Covariates such as birthweight, current bodyweight, GA, PNA, PMA and gender will be evaluated using a stepwise covariate modelling procedure [37].

#### Metabolomics, proteomics

Longitudinal blood sampling from preterm infants offers the potential to explore disease mechanisms and identify potential biomarkers that could be used for diagnosis and prognosis. Proteomic analyses enable identification of host-response proteins in plasma samples. Metabolomic analyses enable the understanding of the disease at molecular level. Analyzing the serum proteome and metabolome are powerful techniques for determining functional changes in the premature neonate in response to disease [38].

Metabolomics and proteomics will be measured in participant’s blood three times where possible and if informed consent is provided for additional blood collection; (1) at moment of onset, (2) 24 hours and (3) 48 hours after onset. Metabolomic biomarkers of the signalling and peroxidised lipid platform will be measured in blood plasma. For proteomic analyses the inflammatory panel from Olink® will be used. These metabolomic and proteomic analyses will be exploratory because they haven't been frequently used in neonatal sepsis research. The aim is to further understand the inflammatory and immunological changes of preterm infants during sepsis with PTX treatment. In order to do so differential metabolomics and proteomic patterns among patients with gram negative sepsis, gram positive sepsis, culture negative sepsis or no infection will be explored. Furthermore, the associations between the metabolomic and proteomic biomarkers and PTX exposure will be explored. Metabolomics levels and patterns will be reported using mass spectrometry, while proteomic levels and patterns will be reported in Normalized Protein eXpression (NPX). Metabolomic analyses require 150 μL plasma and proteomic analyses require 50 μL plasma (400 μL whole blood in total). After centrifugation, the plasma will be harvested, and samples will be stored at −80° before sending for measurement.

### Independent data-monitoring committee

An independent DSMB has been established, which includes a neonatologist, statistician, clinical pharmacologist and pediatrician-infectiologist. The DSMB will monitor the decision regarding dosage increase/decrease. The DMSB will meet after inclusion of the first 6 patients (20% of 30), after 18 (60% of 30) patients and after 30 (100% of 30) and 40 patients (end of study)

### Data management

Data management will be implemented according to Good Clinical Practice (GCP) guidelines. A data management plan is available online (https://dmponline.eur.nl/public_plans). All outcome data will be transferred onto an electric case report form (eCRF, © OpenClinica, LLC and collaborators.), which will be anonymized. An independent monitor (TAPAS Group B.V., Laren, The Netherlands) will be used to control and monitor the study.

### Ethics and dissemination

The patient cannot be included in the study prior to parental/guardian written consent. Partial consent is possible when parents want their child to participate in the dose optimisation trial but are opposed to collecting additional blood samples for PK and Omics. In these patients blood sampling solely concerns residual sample collection. Subjects can leave the study at any time for any reason if parents or guardians wish to do so without any consequences. The investigator and attending physicians can decide to withdraw a subject from the study before the PTX therapy is given, for clinical or other important reasons. The patient will then receive standard care. Data collected up unto that point will be used, if there is are no any objections by parents. The attending physician is also allowed to stop blood sampling for medical reasons. Ethical approval for this study (version 2, 04-10-2019) has been obtained from the regional ethical committee under the reference MEC-2019-0477 on the 11^th^ of December 2019. This trial has been registered on EudraCT (12 July 2019) and on Clinical Trial.gov (1 November 2019). The EudraCT reference is 2019-002020-33 and the ClinicalTrials.gov reference is NCT04152980. Results of the main trial and each of the secondary endpoints will be submitted for publication in a peer-reviewed journal. Authorship of final study outputs will be assigned in accordance with guidelines set out by the International Committee of Medical Journal Editors.

## Discussion

Sepsis remains a major cause of mortality and morbidity in preterm infants [1–3]. Sepsis is associated with an imbalance between pro- and anti-inflammatory reactions and an increased pro-inflammatory reaction, which leads to organ dysfunction and death [39, 40]. Hence, anti-inflammatory drugs have been proposed as a potential treatment for sepsis. One candidate drug is PTX. In adult patients, there is limited clinical benefit of PTX therapy during sepsis. However, in vitro it was observed that the effect of PTX during hyper-inflammation is more pronounced in neonatal cells than in adults cells [41]. In a recent Cochrane review including 6 relatively small studies, Pammi et al. demonstrated that PTX improves survival in neonatal sepsis [15]. Unfortunately, as is the case for most drugs used in neonates, the optimal dosage has not yet been investigated with good quality dose seeking studies for this particular population and indication [8]. This has consequences for both research and clinical practice. In the subsequent placebo controlled trial, a sub-optimal dosage may be compared, leading to sub-optimal findings or increased risk of adverse events [8]. The dosage regimen used in clinical practice might be suboptimal with either under- or overexposure. We propose the first dose seeking study of PTX in preterm neonates suspected of late onset sepsis, in order to optimize sepsis therapy in this vulnerable population. Furthermore, we will perform PK/PD analysis for PTX and its major metabolites following intravenous treatment using a relatively large number of samples. The PTX-trial is a particularly innovative study, since very few studies purposefully attempted to determine the optimal dosage of drugs used in preterm neonates [42–44].

A potential obstacle in sepsis related research could be case selection. This is particularly of importance in dose seeking studies, since dose seeking should be conducted in a population where the signal is more likely. At moment of suspicion, diagnosis and severity of disease are not yet known. The gold standard for diagnosing sepsis in preterm neonates is a blood culture, which could take up to 48 hours [45]. This makes it necessary to integrate certain biomarkers that could objectively be used at moment of suspicion to help and select patients who are most likely to benefit from additional treatment. It is reasonable to assume that patients with (severe) sepsis are most likely to benefit from PTX treatment, while additional treatment given to every suspected neonate could result in overtreatment or in research setting in signal dilution. Therefore, we have incorporated the objective chemical biomarkers IL-6 and CRP in our inclusion criteria, because we these biomarkers reflect disease severity [20]. Besides case selection, timing of sepsis treatment, and in this case especially PTX treatment with its immunomodulatory properties, also matter. Ridings et al. showed that if PTX therapy is delayed until a stage of septic shock is reached, PTX therapy is not effective anymore and could even lead to deleterious effects [31]. If PTX therapy cannot be started timely for any reason patients are excluded from the study.

A potential limitation of the study is the range of selected dosages. We have predefined a series of dosages we aim to test. However, ‘the true optimal dosage’ could be outside the scope of the range we defined. We decided to adapt the infusion time and not the concentration, to avoid high peak serum concentrations (Cmax) for safety reasons. Adjusting the tested dosage by increasing the concentration might be too big of a ‘hit’ to the immune system. If the immune system is hit too hard, the pro-inflammatory reaction might be supressed too severely, which might have harmful effects. However, the current dosage used in clinical practice and research setting, is already proven to have survival benefit [15]. We aim to optimise this dosage and we are not trying to find the maximum tolerated dose. Also we aim to add to the ongoing PROTECT trial, a large placebo controlled randomized controlled that studies the effects of PTX on 2 year handicap free survival (ACTRN12616000405415). It would be synergistic if we could approve the used dosages in that trial.

We assume that once the optimal dosage PTX is evaluated, implementation of this PTX-dose in clinical practice will be feasible and this will help to optimize sepsis treatment in preterm neonates suspected of sepsis. In the Netherlands the online available child formulary ‘kinderformularium.nl’ is a robust construction to facilitate this implementation [46]. This child formulary enables sharing of knowledge regarding dosages, side effects and precautions in pharmacological therapies for children. Moreover, developing a pharmacokinetic model for PTX will enable us to understand the exposure to PTX and its active metabolites to further adapt therapy appropriately in individual patients. Furthermore, longitudinal serum proteome and metabolome measurements will potentially give us an understanding of the functional changes during neonatal sepsis and help us identify potential biomarkers for sepsis diagnosis and prognosis. Also, it will provide us with the possibility to identify patients which could benefit from this specific therapy. Overall this PTX dose finding study aims to improve the treatment of neonatal late onset sepsis in preterm infants and in that way to improve the outcome of these vulnerable patients.

### Patient and public involvement

Care4neo (Association of Parents and Preterm Infants) participate in the study management and are informed about the study aims and study protocol. They supported the research plan and underlined the importance of this project. Furthermore, they were involved in the conceptualization of the informed consent form and information letter.

## Data Availability

The manuscript does not contain any data. The datasets generated during the future study are not publicly available due to privacy legislation but are available from the corresponding author on reasonable request.

## Abbreviations

AUC: Area under the curve
CRP: C-reactive Protein
ED75: Effective dose for 75% of patients
IL-6: Interleukin 6
NEC: Necrotizing enterocolitis
PD: Pharmacodynamics
PK: Pharmacokinetics
PTX: Pentoxifylline
NICU: Neonatal Intensive Care Unit
